# Pilot Feasibility and Preliminary Cost Implications of a Day-Case Head and Neck Surgery Pathway Incorporating ARTISS Fibrin Sealant

**DOI:** 10.7759/cureus.94819

**Published:** 2025-10-17

**Authors:** Ali Abbas, Rishi Shukla

**Affiliations:** 1 Otolaryngology, Oxford University Hospitals NHS Foundation Trust, Oxford, GBR; 2 Otolaryngology, Wycombe Hospital, Buckinghamshire Healthcare NHS Trust, High Wycombe, GBR

**Keywords:** day-case surgery, drain-free surgery, ear nose and throat, ent surgeon, fibrin sealant, head and neck surgery, otolaryngology, parotidectomy, superficial parotidectomy, thyroidectomy

## Abstract

Objective: The objective of this study was to evaluate the feasibility, safety, and preliminary cost implications of implementing a standardized day-case pathway for common head and neck procedures that incorporates ARTISS fibrin sealant (human).

Design: This was a single-center, retrospective pilot service evaluation of consecutive cases.

Setting: The study was conducted at a UK National Health Service (NHS) district general hospital (day surgery unit without inpatient beds).

Participants: Participants included consecutive adults between October 2024 and February 2025 who underwent selected head and neck procedures judged suitable for day-case management under a predefined pathway.

Intervention: A protocolized bundle including meticulous hemostasis, ARTISS applied at closure to promote adhesion and reduce dead space, drain avoidance where feasible (surgeon discretion permitted), structured recovery and explicit discharge criteria, and written or telephone safety-netting.

Main outcome measures: The primary outcome was same-day discharge, and the secondary outcomes included unplanned overnight admission, return to the operating room, 30-day readmission, hematoma or seroma, drain use, and other adverse events. A provider-perspective cost-consequence analysis compared day-case delivery with procedure-specific inpatient length of stay (LOS) estimates from the literature, and one-way sensitivity analyses were performed varying LOS, bed-day costs, and ARTISS price.

Results: Twenty-nine patients underwent day-case head and neck surgery; 25/29 (86.2%; 95% CI 69.4-94.5) were discharged the same day. No returns to the operating room or 30-day readmissions for hemorrhage or seroma were observed (95% upper bound 10.3%). Drains were used in 1/29 (3.4%; 95% CI 0.6-17.2). Base-case modeling indicated bed-day cost savings versus inpatient care.

Conclusions: A standardized day-case pathway that incorporates ARTISS was feasible in this retrospective pilot, with a preliminary favorable safety profile and cost consequences in an NHS setting. As a single-center, non-comparative study, findings are preliminary and cannot isolate the independent effect of ARTISS. Future studies should include multicenter comparative evaluation (ARTISS versus no ARTISS within the same pathway) and formal economic analysis.

## Introduction

Day-case delivery of selected head and neck procedures is expanding in the UK, supported by national day-surgery pathways and service redesign to preserve inpatient capacity [1]. In parotid surgery specifically, multiple centers have shown that day-case parotidectomy is feasible and safe with or without drains, when patient selection, intraoperative technique, and post-discharge support are standardized [2,3]. These findings suggest that a postoperative drain is not required for safe same-day discharge. Moreover, drains can cause discomfort, limit mobility, require removal or monitoring, and drainless pathways are reported to be more convenient and better tolerated by patients [4]. However, whether avoiding drains improves outcomes is less clear.

Fibrin sealants, such as ARTISS (human fibrinogen with low-thrombin concentration), are one strategy to support drain-minimizing pathways by promoting tissue apposition, reducing dead space, and acting as an adjunct to hemostasis [5,6]. This practice is supported by observational comparative studies reporting shorter length of stay (LOS) and cost savings without increased wound morbidity when a fibrin tissue sealant was used as an alternative to drains in parotidectomy and hemithyroidectomy [7-10]. There is also emerging data in lateral neck dissection (LND) indicating that fibrin sealant may reduce drain duration and LOS and, in selected patients, facilitate drainless or even day-case management; other series, however, show no reduction in drain outputs or days, underscoring procedural variation and the need for cautious interpretation [11-13]. Systematic reviews examining the use of fibrin sealants in head and neck soft-tissue surgery indicate modest reductions in drain duration/volume and shorter LOS with fibrin sealants, but certainty is low due to clinical and methodological heterogeneity [14].

In our district general hospital, the day-surgery unit has no inpatient beds, and local policy dictates that drain insertion requires inpatient admission and monitoring. Building on comparative evidence that fibrin sealant-supported drainless pathways can achieve similar safety while allowing same-day discharge [3,15,16], we piloted a standardized day-case pathway across a range of head and neck procedures that incorporates ARTISS as one element of a broader bundle (meticulous hemostasis, layered closure, structured discharge criteria, and post-discharge safety-netting). We report feasibility, safety, and a cost-consequence summary from a consecutive retrospective series; we explicitly avoid claims regarding the independent effect of ARTISS in the absence of a comparator.

## Materials and methods

Study design and setting

This was a single-center retrospective pilot service evaluation conducted at a UK District General Hospital between October 2024 and February 2025.

Ethical considerations

Data collection involved retrospective analysis of routinely collected clinical data; under UK Health Research Authority guidance, Research Ethics Committee review was not required. No identifiable patient information was reported.

Eligibility

Inclusion criteria included adults (≥18 years) undergoing elective head and neck procedures potentially suitable for day-case management, such as hemithyroidectomy, superficial parotidectomy, selective level II/III node excisions, submandibular gland excision, and minor procedures.

Exclusion criteria included significant comorbidity (American Society of Anesthesiologists (ASA) ≥III with decompensation), poorly controlled hypertension, uncontrolled diabetes, or anticoagulation not safely managed per protocol; anticipated difficult airway; and complex neck dissection, such as multi-level dissections, planned flap reconstruction, or combined resections that could prolong procedure time or increase bleeding risk.

Surgical protocol

All procedures were performed under general anesthesia. Hemostasis was achieved using a standardized sequence (head-down positioning and Valsalva to confirm hemostasis), followed by spray application of ARTISS (human fibrin sealant; fibrinogen with low-thrombin) immediately before layered closure to promote tissue adhesion and reduce dead space. A fixed 2 mL pre-filled syringe was used for protocol consistency and to minimize wastage; per manufacturer information, 2 mL covers approximately 100 cm² [5,6]. Deep-layer closure was performed using Vicryl 3/0 parachute sutures and tied within one minute of ARTISS application, followed by manual pressure to the wound for two minutes. Skin closure was completed using subcuticular 4/0 Monocryl and skin glue. Drains were not used routinely.

Postoperative monitoring and discharge

Patients were observed in a day-surgery unit for six hours. Discharge criteria included stable vital signs, hemostasis, tolerance of oral intake, pain control, and patient readiness. Patients not meeting criteria were transferred to the regional tertiary center for admission (no inpatient beds in our facility). Follow-up was arranged via the outpatient clinic or phone.

Outcomes and data collection

The primary outcome was same-day discharge success. Secondary outcomes included postoperative complications (hematoma/seroma, return to operating room, and adverse events), use of surgical drains, 30-day readmission, and follow-up attendance.

Cost analysis

Cost analysis was based on NHS National Cost Collection (2022) [17] estimates of inpatient bed-day cost (£345/day) and ARTISS cost from the supplier (£99.75 per 2 mL). Estimated LOS was based on national guidelines and published literature for each procedure type. Calculated savings reflected avoided inpatient stay costs minus ARTISS product costs.

Statistics

Proportions were reported with 95% CI using the Wilson score method, and zero-event 95% upper bounds were calculated using the rule of three.

## Results

Participants and procedures

Twenty-nine consecutive adults underwent day-case head and neck operations under the standardized pathway. Procedures included superficial parotidectomy (n=14), hemithyroidectomy (n=5), submandibular gland excision (n=2), Sistrunk’s procedure (n=2), branchial cyst excision (n=2), and selective neck dissection (n=4).

Same-day discharge

Same-day discharge occurred in 25/29 cases (86.2%; 95% CI 69.4-94.5). Among these 25 cases, the distribution by procedure was superficial parotidectomy (n=13), hemithyroidectomy (n=3), submandibular gland excision (n=2), Sistrunk’s procedure (n=2), branchial cyst excision (n=2), and selective neck dissection (n=3). Four patients (13.8%) were observed overnight for non-bleeding reasons (e.g., anesthetic observation and patient factors).

Complications and returns 

There were no returns to the operating room and no 30-day readmissions for postoperative hemorrhage or seroma (95% upper bound 10.3% by rule of three). No clinically significant wound complications were recorded during follow-up.

Drains 

Drains were generally avoided because the day-surgery unit has no inpatient beds, and any drain would ordinarily necessitate admission. A drain was used in 1/29 cases (3.4%; 95% CI 0.6-17.2) at the surgeon’s discretion, with the indication documented.

Cost analysis

The cost-benefit of day-case surgery arises from avoided inpatient bed occupancy. The estimated cost of an inpatient bed per day is £345/day [17]. Inpatient lengths of stay for elective ENT procedures were taken from published sources and, where ranges were reported, conservatively rounded up (e.g., 1-2 days treated as 2; 2+ days treated as 3) [17-19]. The cost of 2 mL ARTISS was £99.75.

Table 1 summarizes the assumed inpatient stay, ARTISS cost, and estimated net saving by procedure type. Day-case success by procedure is summarized in Table 2.

**Table 1:** Estimated inpatient stay cost, ARTISS use, and net savings by procedure. LOS, length of stay

Procedure	Assumed LOS (days)	Inpatient bed cost (£345/day)	ARTISS cost (£)	Estimated net saving per case (£)
Superficial parotidectomy	2	690	99.75	590.25
Hemithyroidectomy	2	690	99.75	590.25
Submandibular gland excision	2	690	99.75	590.25
Sistrunk’s procedure	2	690	99.75	590.25
Selective neck dissection	3	1035	99.75	935.25
Branchial cyst excision	2	690	99.75	590.25

**Table 2:** Day-case success by procedure.

Procedure	Number of operations	Successful day case
Superficial parotidectomy	14	13
Hemithyroidectomy	5	3
Submandibular gland excision	2	2
Sistrunk’s procedure	2	2
Selective neck dissection	4	3
Branchial cyst excision	2	2

Cost analysis showed per-patient savings ranging from £590.25 to £935.25, depending on the assumed inpatient LOS. Across 25 successful day-case operations, total estimated savings were £15,791.25. If scaled to 50 procedures per year, projected savings could reach approximately £31,582.50 annually.

## Discussion

In this retrospective, single-centre pilot, a standardised day-case pathway that incorporates ARTISS was feasible across a range of head and neck procedures (parotidectomy, hemithyroidectomy, submandibular gland excision, Sistrunk or branchial lesions, and selective neck dissections), with high same-day discharge and no observed 30-day emergency readmissions. Our observations align with studies reporting that day-case head and neck surgery can be delivered safely with or without drains and with series in which fibrin sealant was used to support drain-minimizing strategies [2-4,7-13,15,16,20,21]. Moreover, this is consistent with the most recent systematic review, which found modest benefits of fibrin sealants on drainage metrics and LOS but graded the certainty of evidence as low due to heterogeneity [14]. Together, these data support the feasibility of drain-minimizing day-case care in selected head and neck procedures.

Finally, our preliminary cost implication signal is coherent with studies [9,10] in which substituting the use of drains with a sealant-supported approach was associated with shorter LOS and cost-saving benefits; however, we treat this as preliminary and contingent on local LOS and costs, reinforcing the need for comparative evaluation and a formal economic analysis.

This study is limited by its small sample size, single-center setting, retrospective design, and absence of a concurrent comparator. The cost analysis is a simplified cost-consequence summary based on assumed LOS ranges and list prices and, therefore, should be interpreted as a directional signal rather than a formal economic evaluation.

Future work should include multicenter comparative evaluation (ARTISS versus no ARTISS within a single, standardized pathway), patient-reported outcomes (comfort and satisfaction), and formal health-economic analysis.

## Conclusions

A pathway-based, drain-minimizing day-case strategy that incorporates ARTISS appeared feasible in this retrospective pilot, with preliminarily favorable safety and cost consequences in an NHS setting. Given the non-comparative design and small sample, generalizability is limited, and no independent effect of ARTISS can be inferred. Multicenter comparative studies with formal economic evaluation are warranted.
